# Cerebrovascular Disease and Cognition in Chronic Kidney Disease Patients

**DOI:** 10.3389/fcvm.2020.00096

**Published:** 2020-06-03

**Authors:** Marius Miglinas, Ugne Cesniene, Marta Monika Janusaite, Arturas Vinikovas

**Affiliations:** ^1^Nephrology and Kidney Transplantation Unit, Nephrology Center, Vilnius University Hospital Santaros Klinikos, Vilnius, Lithuania; ^2^Faculty of Medicine, Vilnius University, Vilnius, Lithuania

**Keywords:** CKD, stroke, silent infarction, leukoaraiosis, microbleed, atrophy, cognitive impairment

## Abstract

Chronic kidney disease (CKD) affects both brain structure and function. Patients with CKD have a higher risk of both ischemic and hemorrhagic strokes. Age, prior disease history, hypertension, diabetes, atrial fibrillation, smoking, diet, obesity, and sedimentary lifestyle are most common risk factors. Renal-specific pathophysiologic derangements, such as oxidative stress, chronic inflammation, endothelial dysfunction, vascular calcification, anemia, gut dysbiosis, and uremic toxins are important mediators. Dialysis initiation constitutes the highest stroke risk period. CKD significantly worsens stroke outcomes. It is essential to understand the risks and benefits of established stroke therapeutics in patients with CKD, especially in those on dialysis. Subclinical cerebrovascular disease, such as of silent brain infarction, white matter lesions, cerebral microbleeds, and cerebral atrophy are more prevalent with declining renal function. This may lead to functional brain damage manifesting as cognitive impairment. Cognitive dysfunction has been linked to poor compliance with medications, and is associated with greater morbidity and mortality. Thus, understanding the interaction between renal impairment and brain is important in to minimize the risk of neurologic injury in patients with CKD. This article reviews the link between chronic kidney disease and brain abnormalities associated with CKD in detail.

## Introduction

Chronic kidney disease (CKD) is a type of kidney disease associated with gradual loss of kidney function, decreased glomerular filtration rate or increased albumin excretion in urine. CKD prevalence is estimated to be 8–16% worldwide (1) and the numbers of CKD patients continue to rise. CKD is associated with increased all-cause and cardiovascular mortality and a number of complications (2). Kidney dysfunction and brain damage is becoming increasingly relevant topic and is worth more focus and attention.

## Risk Factors For Stroke In CKD Patients

CKD in combination with other cardiovascular risk factors accelerates atherosclerosis and increases stroke risk in the predialysis stages. Medial calcifications occur frequently in patients with CKD, independent from the intima calcifications caused by atherosclerosis. Medial calcifications are associated with abnormal cushioning function of blood vessels and increased arterial stiffness. Intimal calcifications are mainly associated with ischemic stroke events, while media calcifications with high pulse pressure contribute to hemorrhagic stroke (3, 4). Endothelial dysfunction and arteriosclerosis each are enhanced by water and sodium retention, uremic toxins, abnormal electrolytes and hyperparathyroidism (5). Some studies also show a link between CKD and cerebral small-vessel diseases (6). Other risk factors for stroke include non-modifiable factors, such as male gender, age, non-Caucasian ethnicity, prior stroke, transient ischemic attack, heart attack and positive family history. Modifiable risk factors include high blood pressure, smoking, diet, obesity, sedimentary lifestyle and atrial fibrillation (AF) (7).

One of the hallmarks of CKD is proteinuria. Patients with proteinuria have a 71% greater risk of stroke compared to those without, as proteinuria is strongly associated with hypertension and other cardiovascular risk factors (8). However, it is not known if interventions which reduce proteinuria are effective in reducing the rates of stroke. Higher urinary albumin-to-creatinine ratio also correlates with higher stroke risk independent of hypertension and estimated glomerular filtration rate (eGFR) (5).

Lower eGFR is associated with higher risk for both ischemic and hemorrhagic stroke (9). There is an ~7% increased relative risk of stroke for every 10 mL/min per 1.73 m^2^ decrease in glomerular filtration rate, and the finding is consistent across major stroke subtypes (10). For individuals with end-stage renal disease, far less is known regarding the stroke risk factors. The hemodialysis procedure itself may increase the risk of stroke. Drastic hemodynamic changes, high variability of blood pressure, dialysate and anticoagulants, vascular access, dialysis amyloidosis, vascular calcification, and years on dialysis, may trigger both ischemic and hemorrhagic strokes (11). Among 151 Japanese hemodialysis patients with acute stroke, almost a half brain infarcts and more than one third of brain hemorrhages occurred during or <30 min after start of dialysis session; the remainder occurred at other unspecified times (12, 13). An analysis of ~21,000 US dialysis patients found that stroke rates reached a peak during the first 30 days after dialysis initiation (13). Thus, dialysis initiation constitutes the highest stroke risk period.

Atrial fibrillation (AF) is another significant stroke risk factor common in CKD patients with an estimated prevalence ranging from 3.5 to 27% depending on AF type (14). CKD is often associated with hypertension and high atrial pressure as well as activation of the renin–angiotensin–aldosterone system (RAAS). Angiotensin II can promote atrial fibrosis, increase atrial pressure and modulate ion channels, all of which are involved in structural and electrical remodeling of the atria, thus resulting in AF (15). AF and CKD combination leads up to 5-fold increase in the stroke risk (16).

## Clinical Relationship Between Chronic Kidney Disease, Stroke and Stroke Outcomes

Stroke is one of the most frequent neurological conditions and the leading cause of death. As patients with CKD have more risk factors for atherosclerosis, it is estimated that the risk for developing cardiovascular diseases, including stroke, is 5–30 times greater than in the general population (2). The prevalence of coronary artery disease increases as kidney function declines (~40% in patients with end stage kidney disease) (3, 17). The risk of stroke in CKD patients is particularly high. The annual incidence of stroke is 15.1% in hemodialysis and 9.6% in CKD patients. In comparison the annual incidence of stroke is 2.6% in patients without CKD (18). Stroke rate in peritoneal dialysis patients is less well-characterized. Wang et al. compared a general population group (669,773 non-dialysis individuals) with hemodialysis (74,192 patients) and peritoneal dialysis (5,974 patients) groups. PD patients had a lower risk of hemorrhagic stroke (HR, 0.75; 95% CI, 0.58–0.96), while there was no significant difference in the risk of ischemic stroke between PD and HD patients (19). CKD significantly worsens stroke outcomes. Patients with stage 3–5 CKD have worse survival prognosis, greater neurological deficit following ischemic zone and poor functional outcomes following stroke. CKD patients may have up to 49% greater risk of neurological deterioration during their hospital stay, defined as at least a 2-point increase in the National Institutes of health (NIH) Stroke Scale score, a 138% greater risk of in-hospital mortality and a 25% greater risk of a Modified Ranki Scale (mRS) score of 2 or more at discharge than patients without CKD (20, 21). Similarly stroke in end-stage renal disease patients on hemodialysis is associated with inferior prognosis, especially in the case of hemorrhagic stroke (11, 22). Thus, chronic kidney disease determines poorer vitality and functional outcomes after hemorrhagic and ischemic strokes.

## Functional Brain Damage In Patients With Chronic Kidney Disease

Recently mild cognitive impairment (MCI) has garnered a scientific interest in the nephrology community (23). CKD is an independent risk factor for cognitive decline (24). MCI starts early in the course of the CKD and parallels kidney function decline (25). A reasonable strategy to prevent this is to control cardiovascular risk factors according to current standards of care. Although cognitive dysfunction is obviously very relevant, prevalence, assessment and exact clinical implications of it in the setting of CKD are still being defined. Furthermore, data on interventions slowing cognitive decline in CKD patients are very limited. On the other hand, the kidney-brain relationship is bidirectional, as the kidney function declines more rapidly in patients after brain infarction (26).

Cognitive impairment affects quality of life (27). Cognitive dysfunction has been associated with poor health literacy, low adherence to medication, and greater morbidity and mortality (28). A recent study by van Zwieten et al. showed that in hemodialysis patients, those impaired in at least 1 cognitive domain had 77% higher adjusted hazard for mortality compared with those with no impairment. This association was dose-dependent as mortality risk increased with the number of domains impaired. These associations between cognition and mortality persisted after adjustment for confounders (29). The pattern of cognitive impairment in CKD is not well-defined. Interestingly, a study of 676 adult hemodialysis patients from 20 centers in Italy evaluated the prevalence and patterns of cognitive impairment across five domains of learning and memory, complex attention, executive function, language and perceptual-motor function. In this study cognitive function has been assessed using a neuropsychological battery of 10 tests. The study showed that cognitive impairment is extremely common in hemodialysis patients with an estimated 71.1% of patients being impaired on at least one domain, and patients often experience multiple deficits simultaneously with 45.2% of patients impaired on two or more domains (30).

The data supporting CKD-induced brain injury consist of epidemiological associations, expert opinion, and few studies. Brain lesions can be assessed either by radiological methods, which provide largely structural information, or validated neurocognitive tests with a functional information (31).

**Figure 1:**
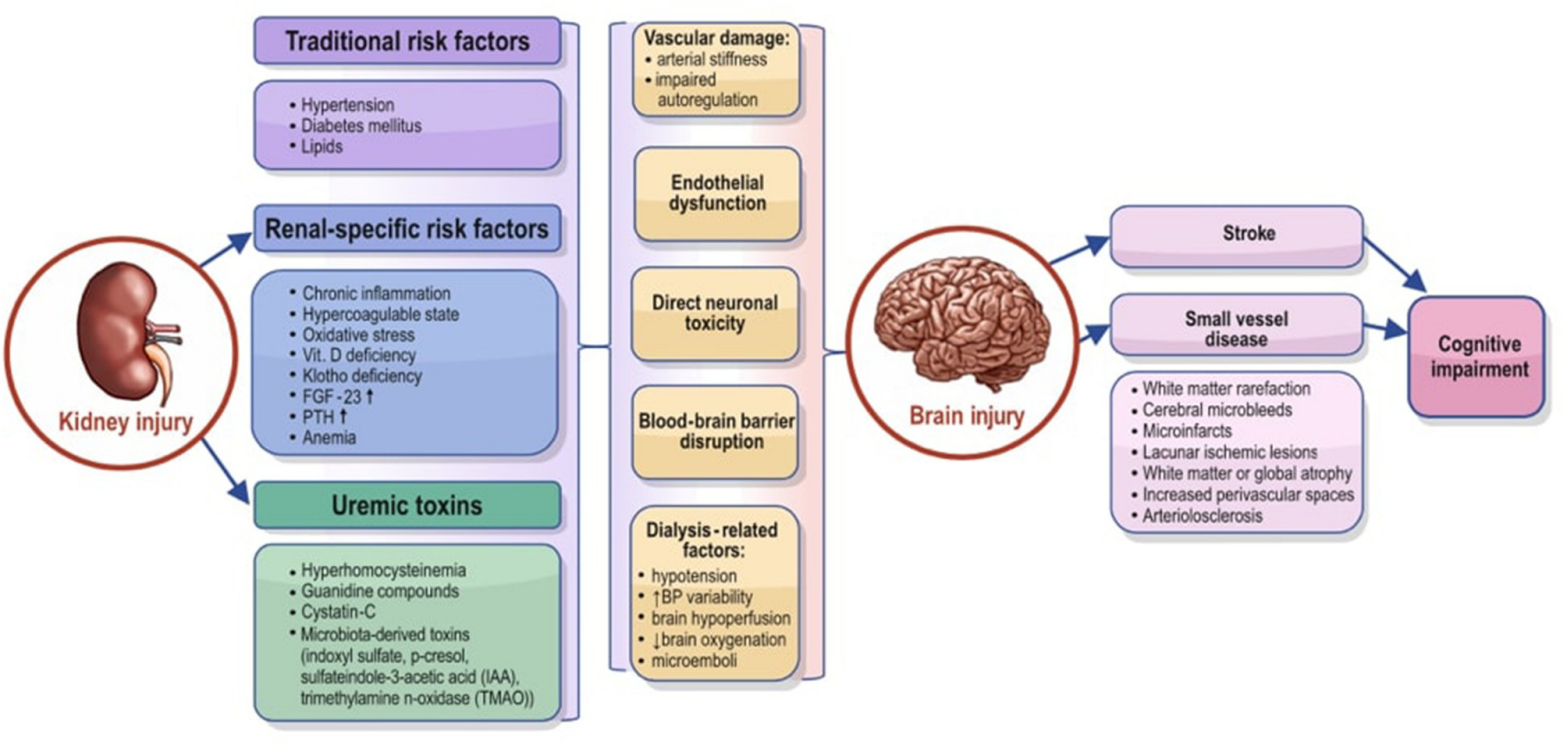
Possible mechanisms that can explain the association between chronic kidney disease and brain injury. FGF-23, fibroblast growth factor 23; PTH, parathyroid hormone; IAA, sulfateindole-3-acetic acid; TMAO, trimethylamine n-oxidase.

The pathophysiology of cognitive dysfunction in CKD is very complex and encompasses endothelial dysfunction, vascular calcification and other vascular structural changes, impaired autoregulation, and various humoral factors specific to kidney disease. Possible mechanisms of brain injury in the setting of CKD are summarized in Figure 1.

Various hypotheses have been proposed to explain kidney-brain interaction. “Vascular hypothesis” implies that impaired cerebral hemodynamics in CKD may lead to cognitive decline (32). Brain has a low vascular resistance system (with local autoregulation), which allows continuous high-volume blood flow. It makes this organ vulnerable to microvascular injury caused by systemic hypertension (33). Accelerated arteriosclerosis in CKD may impair cerebral blood flow autoregulation. This causes microvascular injury incurred by transmission of central aortic pressure into cerebral capillaries. In addition, cerebral small vessel disease due to increased artery stiffness is believed to contribute to MCI in CKD. However, there are studies showing that CKD is associated with dementia independently of cerebral small-vessel disease (34).

“Neurodegenerative” hypothesis relates cerebrovascular injury in CKD to accumulating uremic toxins, disrupted blood-brain-barrier integrity (BBB), neurotransmitter derangement, and disturbed drug pharmacokinetics. The BBB and the blood-cerebrospinal fluid barrier (BCSFB) are paramount to keep central nervous system stable from fluctuating blood composition. These barriers consist of tightly connected cerebral endothelial cells, astrocytes and other components. This structure allows BBB and BCSFB to control transportation of various proteins and nutrients between central nervous system and the blood. Any BBB or BCSFB disorder or injury determines increased transportation of inflammatory cells and proteins into the brain tissue (35). BBB permeability in CKD is not well-described. Phosphate, indoxyl sulfate and the soluble receptor for advanced glycation end products (sRAGE) have been shown to be toxic to endothelial cells. The mechanisms involved include reactive oxygen species production, junctional breakdown of the myosin light chain via MEK-ERK-mediated phosphorylation and inflammatory response induction (36–38). Interestingly, there are rare clinical observations of contrast-induced encephalopathy (CIE) in patients with renal impairment. CIE is an acute neurological disturbance after the intra-arterial iodinated contrast medium administration (39). Extravasation of contrast from the brain capillaries is suggestive of disruption of BBB integrity in CKD patients. There are profound changes in the structure and function of gut microbiota in CKD as well (40). Urea and other metabolic waste products diffuse into the gut lumen and induce changes in the microbiota, thus leading to generation of proteolysis waste products, such as indoxyl sulfate (IS), p-cresyl sulfate (p-CS), indole-3-acetic acid (IAA), trimethylamine n-oxidase (TMAO) etc. (“gut-derived uremic toxins”) (41, 42). The increased intestinal toxins production further contributes to uremia. These substances may activate the intestinal mucosa immune system and disrupt intestinal permeability, with translocation of bacterial products into the host circulatory system, thereby promoting the generation of inflammatory factors, ROS production, fibrosis development and apoptosis. It has been shown that a higher serum IS level is associated with poor executive function in the early stage of CKD (43). IS-mediated immune dysfunction provokes vascular endothelial cell damage in ESRD patients (44). TMAO metabolite has been proposed as a potential surrogate marker to detect early vascular risk in patients with CKD (45). Thus, “gut-derived uremic toxins” induce vascular damage and play a crucial role in the pathogenesis of cerebrovascular disease.

There are various other kidney-specific factors involved in pathophysiology of cognitive decline in patients with renal impairment. Secondary hyperparathyroidism can potentially interfere with neurotransmission by increasing calcium levels in the brain (46). Anemia and malnutrition in CKD may also impair oxygen delivery of to the brain and affect brain metabolism (47). In end-stage renal disease patients dialysis itself leads to cognitive decline through rapid changes in blood pressure causing brain hypoperfusion, microemboli, edema, or cerebral hemosiderin deposition (48). Intra-dialytic hemodynamic instability may affect cerebral oxygen delivery. A useful parameter in this setting is brain oxygenation, which reflects both oxygen delivery and consumption. One suitable brain oxygenation measurement method is near-infrared spectroscopy (NIRS). It allows a continuous, non-invasive measurement of frontal lobe tissue oxygenation. Cerebral NIRS is based on the fact that infrared light can penetrate the skull (49). Hemoglobin oxygen saturation changes are determinated from the different wavelength absorption of near-infrared light by oxygenated and deoxygenated hemoglobin (50).

The effect of various treatment strategies on cognitive function in CKD patients has been investigated. This includes more frequent or nocturnal dialysis and other (51). However, only few intervention trials have been performed (52). New dialysis strategies with more efficient protein-bound metabolite clearance is a promising strategy (53). High dose vitamin B treatment of hyperhomocysteinemia did not improve cognition a randomized placebo-controlled trial of CKD patients (54). Treating renal anemia with erythropoietin may potentially improve cognitive function, but cognition was not specifically addressed in erythropoietin trials. On the other hand, erythropoietin use has been shown to be associated with an increased risk of stroke, which itself is a major risk factor for cognitive decline (55). A recent study failed to confirm an association of anemia and cognitive decline (56). On the other hand, kidney transplantation is associated with long-term cognitive improvement in dialysis patients (57, 58).

Effect of lower blood pressure levels on age-related cognitive decline in patients with and without CKD has been investigated in one large trial (Systolic Blood Pressure Intervention Trial). Recently published SPRINT MIND study shows that intensive blood pressure control significantly reduces the risk of mild cognitive impairment (HR, 0.81; 95% CI, 0.69–0.95) in whole cohort and in those with CKD (HR, 0.79; 95% CI, 0.56–1.11) (59). The role of vitamin D (60), FGF-23 levels (61), klotho deficiency (62), exercise (63) is still being debated. CKD-related neuroinflammation is an attractive target for prevention of cognitive impairment (64). It has been shown that an anti-IL-1β antibody canakinumab (65), and the anti-inflammatory drug colchicine (66) favorably affect brain function in patients with cardiovascular disease. Cognitive-behavioral interventions may be very effective (67). Cognitive stimulation therapy, which involves discussions by trained staff, improved cognition in other patient populations (68) and has to be studied in patients with CKD as well. A supervised exercise should be encouraged to prevent cognitive decline in CKD patients (69).

Further research and intervention strategies are required to explore the cognitive decline in CKD.

## Neuroimaging and Brain Lesions In Chronic Kidney Disease

### CKD and Cerebral Microbleeds

Cerebral microbleeds (CMBs) or microhemorrhages are 5–10 mm size round, homogenous, hypointense foci that can be visualized by magnetic resonance (MR) susceptibility-weighted (SW) imaging. Histopathologically, CMBs represent hemosiderin-laden macrophages, hemosiderin granules and different kinds of vascular damage (e.g., microaneurysm, dissection in the wall of a distended vessel) (70). In the ethiopathological classification, CMBs are assigned to a hemorrhagic group of small-vessel disease lesions. The main pathologic processes that can affect small vessels of brain are arteriolosclerosis and/or cerebral amyloid angiopathy (CAA). The anatomic localization of CMBs reflects the underlying pathological vessel damage—lesions in cortical-subcortical regions are seemingly caused by β-amoyloid deposition (cerebral amyloid angiopathy), while the ones in deep brain regions are presumed to be the result of arteriolosclerosis (71). CMBs are a predictive factor for a future risk of overt cerebrovascular disease. The presence of microbleeds, especially multiple, predict future stroke (first event including)—the ones located in regions not typically affected by cerebral amyloid angiopathy are associated with an increased risk of both ischemic and hemorrhagic stroke, while CMBs in regions, where CAA is highly prevalent, is associated with a higher risk of intracerebral hemorrhage (72–74).

CMBs are more frequently observed in subjects with renal dysfunction. Previously neurologically healthy adults with CKD have been investigated in a recent study. CMBs were spotted as the patients underwent MRI as a part of health screening program. The results have shown a significant association between the decreased kidney function and the presence of CMBs. A decrease in GFR level was associated with an increase of the number of lesions (*p* for trend = 0.003) (72, 75). The above mentioned studies have limitations that hinder the generalizability of the results, like small sample size, study population with specific diseases. Conversely, there are data which do not support the association between the renal function decline and CMBs. These data must be evaluated critically because of the lack of study statistical power, differences in ethnicity and other limitations (76).

There are recent data on CMBs in patients on dialysis as well. Hemodialysis patients with no stroke history had been prospectively followed-up until they died or were transplanted. Study showed CMBs being a strong predictor for a future intracerebral hemorrhage, but not a cerebral ischemia (77, 78). The impact of CKD on CMBs may differ between patients with or without diabetes mellitus. CKD in patients without diabetes has two to four times greater risk of CMBs (79).

From mechanistic perspective there are recent data pointing to the role of disruption of the blood–brain barrier in the genesis of CMBs in CKD (80). The paper reports an increased prevalence of cerebral microhemorrhages in CKD mice. Interestingly, microhemorrhages appeared to be independent of hypertension. CKD increased brain microbleeds in both hypertensive and non-hypertensive mouse models. Uremic serum disrupted the cultured brain endothelial monolayer due to disarranged actin cytoskeleton and decreased tight junction proteins in the cells. These findings support a mechanistic role for uremic toxins affecting BBB permeability and promoting microhemorrhages.

In summary, different studies suggest that the decreased kidney function is a risk factor for CMBs, while the CMBs are considered to be an independent risk factor for overt cerebrovascular disease. Therefore, renal function can serve as a potential alternative predictor for cerebrovascular events. More studies are needed to determine if moderation of kidney disease can prevent the occurrence and progression of CMBs.

### CKD and Silent (Asymptomatic) Brain Infarction

Silent brain infarction (SBI) is a cerebral infarction that is visible on brain imaging but does not cause any clinical symptoms (81). SBI is a lacunar infarction that is caused by the occlusion of small cerebral arteries. Infarcts are detected by CT or MRI as focal lesions with roughly the same intensity as cerebrospinal fluid. However, sensitivity of infarcts detection is better for MRI compared to CT, due to improved MRI techniques. The prevalence of SBI varies and depends on the population studied and imaging technique used (82).

Clinical studies suggest that SBI is a strong predictor for stroke (83), moreover some investigations indicate SBI as a risk factor of dementia and mild cognitive decline. Although arterial hypertension is the strongest risk factor of SBI, there are data showing to the connection between CKD and SBI (84, 85). General population study in Japan found a close association between SBI and eGFR in healthy adults, where the prevalence of SBI increased with advancing stage of CKD (6). Furthermore, other investigations show that vascular events can be predicted by SBI in end-stage renal disease patients (85, 86). A cross-sectional study involving 1,937 neurologically normal subjects with mild CKD revealed a significant association between CKD silent brain infarction, independent of other risk factors (33). In summary, the significance of SBI in kidney disease population remains to be defined. Future investigations should identify factors associated with accelerated SBI progression and conversion from asymptomatic cerebrovascular disease to symptomatic stroke.

### Clinical Relationship Between Chronic Kidney Disease and Cerebral Atrophy

There is a wide range of neurological pathologies which are related to CKD, including brain atrophy. Even in non-dialysis CKD patients, imaging studies consistently demonstrate a greater evidence of cerebral atrophy than in the general population.

Mechanisms that connect brain atrophy and renal injury have not been fully explored, but several hypotheses might explain the association between both processes. Previous studies have shown that increasing small vessel disease is associated with cerebral atrophy (87). There are several explanations for an increased prevalence of brain atrophy in CKD population. First, brain has a low vascular resistance system (with local autoregulation), which allows continuous high-volume blood flow. It makes this organ vulnerable to microvascular injury caused by systemic hypertension (88). Second, endothelial dysfunction might be an influential cofactor for damaging processes of both organs. The renin-angiotensin-aldosterone system should also be considered as a cofactor in both CKD and small vessel disease degenerative changes, as it affects BP regulation, vasoconstriction, thrombosis and vessel wall damage (89).

Yakushiji et al. investigated the relationship between kidney function and subclinical age-specific cerebral atrophy in adults with no previous history of neurological disorders. They showed that decreased GFR is significantly associated with a substantial degree of cerebral atrophy (89). In one of studies brain atrophy measured by voxel based morphometry (VBM) has been compared between CKD and healthy patients. The results showed significantly lower gray matter volume in those with ESRD. Similar investigation has been done later and ESRD patients in comparison with healthy adults showed association between diffusely decreased gray matter volume and increased serum urea. Duration of dialysis was negatively associated with changes in white matter volume (90, 91). There are data indicating higher prevalence (even at younger age) and more rapid progression of brain atrophy in dialysis patients (92). ESRD patients on dialysis show significant cerebral atrophy correlation with longer dialysis duration (93). Thus, clinical significance of cerebral atrophy in kidney disease has to be investigated further. There are studies demonstrating the correlation between brain atrophy and executive dysfunction in CKD patients (94).

### CKD and White Matter Lesions (Leukoaraiosis)

White matter lesions (WMLs) are areas of demyelinated cells found in the white matter of the brain. Moreover, WMLs are associated with increased risk of stroke, cognitive decline, and dementia. Recently, WMLs has been studied more, to clarify their link with CKD (95), because CKD is known as a risk factor for cerebrovascular disease (96). Rotterdam Scan Study involving 484 participants, showed that people with decreased eGFR had less deep white matter volume and the volume of deep white matter lesions was significantly increased (97). Similarly, the Northern Manhattan Study (NOMAS) revealed that eGFR of 15–60 mL/min/1.73 m^2^ was significantly linked to an increased volume of WMLs (β 0.322 [95% CI 0.080–0.564]) after adjusting for cardiovascular risk factors) (96). Thus, patients with CKD show disproportionate high levels WMLs on imaging. These findings are clinically important because WMLs are not benign. They may clinically present as cognitive impairment with the subsequent development of dementia.

### Therapies and Interventions for Stroke Prevention in CKD

Understanding the risks and benefits of established stroke prevention treatments is vital in patients with CKD, especially in those on dialysis. Especially, post-stroke management with anticoagulants, antiplatelet agents and lipid-lowering agents to prevent recurrent strokes presents the risk of undesirable side effects in CKD patients with reduced renal clearance.

Stroke risk in CKD, similar to the general population, increases with higher systolic blood pressure (BP). Thus, blood pressure lowering is an attractive approach for both primary and secondary stroke prevention (98). However, the BP targets for stroke prevention in CKD population remains debated. The effect of intensive blood pressure reduction (systolic BP <130 mm Hg vs. 130 to 149 mm Hg) in patients with recent lacunar stroke with and without CKD has been recently reported in a secondary analysis of the SPS3 (Secondary Prevention of Small Subcortical Strokes) trial (99). CKD patients with a history of lacunar stroke showed a 50% increase in risk of recurrent stroke. There was no difference of intensive BP lowering on death, stroke, myocardial infarction, or intracranial hemorrhage between patients with and without CKD. This finding is consistent with the results of the SPRINT (Systolic Blood Pressure Intervention Trial), where the advantageous cardiovascular outcomes of intensive BP lowering persisted in patients with CKD (100). In the China Stroke Primary Prevention Trial (CSPPT) a time-averaged systolic BP of ≤ 135 mmHg showed reduced risk of total first stroke and ischemic stroke in mild-to-moderate CKD patients (101). However, the effects of intensive blood pressure BP lowering in terms of renal protection or harm remain controversial (102). Treatment-induced albuminuria change is of utmost importance (103). A meta-analysis of 32 randomized controlled trials showed up to 29% risk reduction of cardiovascular endpoints for each 10% decrease in albuminuria levels (104). In the line with these findings Kidney Disease Improving Global Outcomes (KDIGO) clinical practice guidelines recommend a BP target of <140/90 mmHg in those with albumin excretion <30 mg/24 h and a BP goal of <130/80 mmHg in those with albumin excretion >30 mg/24 h (105). The latest ESC/ESH guidelines recommend BP reduction to <140/90 mmHg and toward 130/80 mmHg (but systolic BP not below 130 mmHg) (106). Overall, these BP targets seem to be reasonable for both primary and secondary stroke prevention in CKD patients. Renin angiotensin system (RAS) blockers not only lower the risk of cardiovascular events but delay the progression of CKD itself in this population (107). However, treatment has to be adjusted according to its impact on renal function and electrolytes, especially in patients with advanced CKD (108). RAS blockers are clearly beneficial for secondary prevention of stroke in CKD. As it has been shown in Perindopril Protection against Recurrent Stroke (PROGRESS) study there was a 35% reduction in the recurrent stroke risk in kidney disease patients with a history of cerebrovascular disease (109). Perindopril prevented one stroke among every 11 patients treated over 5 years.

Hypertension appears to be an important mediator of hyperuricemia as a risk factor for stroke. REGARDS (Reasons for Geographic and Racial Differences in Stroke) study confirmed that hyperuricemia is associated with higher risk of ischemic stroke. Mediation analyses showed that indicators of hypertension severity are statistical mediators of the hyperuricemia-stroke association (110). The study supports the joint roles of hyperuricemia and hypertension severity with increasing ischemic stroke risk. A recent meta-analysis demonstrates that hyperuricemia-related cardiovascular risk (including stroke) can be modified by treating hyperuricemia and gout with xanthine oxidase inhibitors (111). In Febuxostat for Cerebral and CaRdiorenovascular Events PrEvEntion StuDy cerebral, cardiovascular, renal events and all deaths were significantly reduced in the febuxostat group compared with non-febuxostat treatment [hazard ratio (HR) 0.750, 95% CI 0.592–0.950; *P* = 0.017] (112). Both febuxostat and allopurinol showed similar rates of cerebral and cardiovascular events in the CARES trial. But, on a cautionary note, all-cause mortality resulting from cardiovascular deaths (sudden cardiac death) has been reported to be higher with febuxostat (113). In addition, uric acid may show neuroprotection in the acute phase of stroke due to its antioxidant properties (114).

Sodium-dependent glucose transporters (SGLTs) proteins may be a viable therapeutic target in ischemic stroke in CKD patients. Interestingly, SGLTs are regionally increased in ischemic brain especially in the penumbral regions (115). Large cardiovascular outcome trials have demonstrated the beneficial effects of these agents beyond glycemic control. Indeed, sodium-glucose cotransporter-2 (SGLT2) inhibitors show a great efficacy in treating diabetes, cardiac disease, stroke, and kidney disease (116). Recent trial data suggest that SGLT2 inhibitors may be vasculoprotective even in patients with non-diabetic kidney disease (117). This area is being extensively explored further (118).

CKD is a coronary heart disease equivalent for future cardiovascular events, with risk exceeding that of diabetes mellitus (119). Thus, KDIGO recommends statin treatment for CKD patients over 50 years of age without any individual risk calculation (120). Lipid lowering with statins is effective at reducing the risk of stroke in 3–4 stage CKD patients (121). Current data do not support lipid lowering for dialysis patients with inflammation and/or malnutrition or treatment-naive dialysis patients (122). LDL targets for cerebrovascular protection in CKD patients deserve a further research.

Traditional anticoagulation guidelines with warfarin or novel oral anticoagulants (NOAC) for the general population for thromboembolic stroke prevention in AF may not be applied for advanced kidney disease or dialysis patients. Warfarin in patients with both ESRD and atrial fibrillation are associated with an increased risk for stroke, possibly due to accelerated vascular calcification occurring as a result of vitamin K antagonism (123). This risk is greatest in warfarin users who do not receive in-facility INR monitoring (14). Due to NOAC renal elimination pathway there is a potentially greater risk for bleeding associated with the prolonged half-life. The dosing recommendations for patients with renal impairment differ depending on the NOAC, with some of the NOACs requiring dose reductions based solely on renal function and others taking additional criteria into consideration (124). CKD patients have increased both thromboembolic and bleeding risk, limiting pharmacotherapy options. Therefore, evidence-based treatment is offered less often in CKD population due to fear of increased risk of side effects of the utilized antithrombotic agents.

According to American Heart Society American College of Cardiology and American Heart Association 2019 atrial fibrillation guidelines NOACs are recommended over warfarin except in patients with moderate to severe mitral stenosis or a prosthetic heart valve, although kidney and liver function should be tested before initiation of NOAC and evaluated annually. For patients with AF and moderate-to-severe CKD with an elevated CHA2DS2-VASc score, treatment with reduced doses of direct thrombin or factor Xa inhibitors may be considered (e.g., apixaban, rivaroxaban, edoxaban, or dabigatran). In AF patients with a CHA2DS2-VASc score ≥2 in men or ≥3 in women and a creatinine clearance <15 ml/min or who are on dialysis, it is reasonable to use warfarin or apixaban for oral anticoagulation (125). The 2018 European Heart Rhythm Association practical guide states that compared with warfarin, all four NOACs showed consistent efficacy and safety in patients with mild to moderate CKD compared with non-CKD patients in the respective subgroup analyses of pivotal NOAC trials. Rivaroxaban, apixaban, and edoxaban (but not dabigatran) are approved in Europe for the use in patients with severe CKD (Stage 4, CrCl of 15–29 mL/min) (126). In the US (but not in Europe) apixaban twice daily is currently approved in chronic, stable dialysis-dependent patients.

There are limited data on the NOAC use in patients with advanced kidney disease. It is important to note that patients with calculated creatinine clearance (CrCl) <25–30 mL/min (Cockcroft-Gault), including those on dialysis, were excluded from the major NOAC trials. However, results from an ARISTOTLE *post-hoc* subanalysis in patients with stage 4 chronic kidney disease (CKD) (CrCl 15–29 mL/min) showed (*N* = 269) that the effect of apixaban vs. warfarin on the primary efficacy outcome of stroke or systemic embolism was similar in each kidney function category, over a median 1.8 years follow-up (<30 mL/min vs. ≥30 mL/min; *P* interaction = 0.50). Major bleeding event rates were numerically lower in patients receiving apixaban compared with warfarin, irrespective of kidney function category (CrCl <30 mL/min vs. ≥30 mL/min), which was consistent with the overall population. In patients with CrCl <30 mL/min, the median apixaban drug exposure was 5,512 ng/mL^*^h in 12 patients assigned the 5 mg dose and 2,792 ng/mL^*^h in 19 patients assigned the 2.5 mg dose (127).

A retrospective cohort study of Medicare beneficiaries comparing ESRD patients receiving warfarin and NOAC apixaban found that patients receiving standard-dose apixaban (5 mg) had a lower risk of stroke/embolism than those receiving low-dose apixaban (2.5 mg) and warfarin. Standard-dose apixaban was associated with a lower risk of death than that observed with low-dose apixaban and warfarin, and there was a lower risk of major bleeding with apixaban than with warfarin (128). A recent study selected patients with AF and stage 4–5 CKD on rivaroxaban or warfarin from the Optum® Deidentified Electronic Health Record Database. No statistically significant difference in the risk of ischemic stroke/systemic embolism (ISSE) or major bleeding was found between rivaroxaban- and warfarin-treated patients. Authors conclude, that rivaroxaban appears to be a reasonable alternative to warfarin for ISSE prevention in stage 4–5 CKD patients with AF (129). There is another interesting report from retrospective propensity score matched, US claims database of just below 50 000 apixaban, dabigatran, rivaroxaban or warfarin users due to atrial fibrillation and CKD stages 4–5. In patients CKD stage 4–5 the use of NOACs reduced the risk of stroke without increasing bleeding. In patients not on dialysis, compared to warfarin, NOACs had similar risks of stroke, and apixaban had a lower risk of bleeding (130). Recently NOACs (apixaban, dabigatran and rivaroxaban) were compared to vitamin K antagonists (VKAs) by eGFR group (≥60, 30–59, <30 ml/min/1.73 m^2^). In this population-level, retrospective cohort study from Canada 27,552 new NOAC users were matched to 27,552 new VKA users. The anticoagulation was indicated for the prevention of thromboembolism in atrial fibrillation or deep venous thrombosis. NOACs compared to VKAs were associated with lower risk of cardiovascular events or mortality. Interestingly, this association was strongest among those with reduced eGFR. No interaction of eGFR and anticoagulant class was detected for the hemorrhage outcome (131).

Several randomized trials are currently underway to definitively assess the treatment effects of NOACs compared with warfarin for stroke prevention in patients with advanced kidney disease (132, 133). In the meantime, clinicians should continue to weigh the risk of stroke vs. bleeding before prescribing NOACs in this medically complex population.

Thrombolysis with tissue plasminogen activator (tPA) is a clinical challenge in CKD patients. This patient group has such subclinical manifestations as transient infarcts, lacunar infarcts and microbleeds. ESRD patients are even more complex because of comorbidities, such as atrial fibrillation, anemia, diabetes mellitus and hypertension (134). Furthermore, cardiovascular complications and venous thrombosis that mainly dominate at the early stages of CKD suggest a prothrombotic state, while procoagulant state persists with episodes of bleeding due to the strong association of uremic toxins with platelet dysfunction at advanced stages of CKD (135). CKD also interferes with contrast tomography imaging, increases bleeding risk and alters thrombolysis efficacy. Thrombolysis in these patients is associated with high rate of in-hospital mortality and unfavorable outcomes (136). CKD patients exhibit high levels of endogenous circulatory tPA, thrombotic mediators, endothelial damage, platelet dysfunction, and infiltration of inflammatory cells into the brain. All these cumulatively could aggravate the bleeding complications after recombinant tPA administration in stroke-CKD patients. Non-fibrinolytic mechanisms of tPA suggest that CKD patients are highly vulnerable to tPA-induced complications, even at an early stage of CKD (137). Furthermore, the effectiveness and safety of thrombolytic therapy for acute ischemic stroke are unknown for patients with mild to moderate CKD. Several small studies did not find an increase in intracranial hemorrhage in patients with CKD stages 3–4 (138). In patients with advanced CKD, however, an increased risk of symptomatic intracerebral hemorrhage and mortality has been reported (139). Overall, major stroke guidelines do not preclude use of intravenous thrombolytic agents in eligible CKD patients, even in those on dialysis (140). The American Heart Association/American Stroke Association 2018 guidelines on early management of patients with acute ischemic stroke state that in patients with end-stage renal disease on hemodialysis and normal aPTT, IV alteplase is recommended (Class I; LOE C-LD). However, there is a warning, that those with elevated aPTT may have elevated risk for hemorrhagic complications.

Clinical guidelines advocate the use of antiplatelet therapy for the prevention of ischemic stroke in high risk patients (11, 141). However, the balance of benefits and risk remains unsettled in subjects with CKD (142). A review of more than three decades of investigation identified very few studies to inform physicians on the use of antiplatelet agents in patients with CKD (143). There seems little benefit from treatment with antiplatelet agents, whilst risk of bleeding may increase. A recent meta-analysis included four randomized controlled trials and four observational studies of 38,341 CKD patients, including stage 3–5 CKD and those on dialysis (144). And again there was no preventive effect on CVD events. On the other hand, there was not enough evidence to not use antiplatelets in CKD or ESRD patients. Some evidence supports antiplatelet therapy efficacy for ischemic stroke prevention in hypertensive 3–4 stage CKD patients (145). Antiplatelet therapy may prevent major vascular events in 5HD stage CKD (146). To certain extent poorer outcomes in CKD patients in some studies may explained by antiplatelet hyporesponsiveness in these patients (147). There is acquired thrombocytopathy characterized by decreased aggregation of platelets in response to stimuli in CKD. It has been proposed that renal patients have deficient platelet α-granula release, dysregulated arachidonic acid and prostaglandin metabolism, and reduced ADP release (148). Overall, the guideline recommendations differ regarding antiplatelet use for primary prevention in CKD (149). NICE and KDIGO guidelines do not indicate aspirin for primary prevention. ACC/AHA/ASA recommend aspirin for primary prevention only if eGFR is 30–45 ml/min/1.73 m^2^. These discrepancies may lead to great practice variations. In the UK more than 3 million people with CKD and no cardiovascular disease are not prescribed aspirin but ~1 million receive aspirin for primary prevention (150). The ongoing Aspirin To Target Arterial events in Chronic Kidney Disease (ATTACK) study will attempt to assess the antiplatelet effects for risk reduction of major cardiovascular events in patients with kidney disease (151).

The guidelines consistently recommend the antiplatelet use for secondary stroke prevention in the general population (152). CKD patients are no exclusion, because the stroke recurrence rates in this population are very high (99). However, dual antiplatelet therapy (DAPT) seems to be not so beneficial for CKD patients in this setting (153). Renal function considerations should be made in modern antiplatelet treatment with P2Y12 receptor inhibitors (154). The antiplatelet effect of clopidogrel is decreased in patients with CKD. In a *post-hoc* analysis of the CHARISMA trial (Clopidogrel and Aspirin vs. Aspirin Alone for the Prevention of Atherothrombotic Events) diabetic CKD patients on clopidogrel showed increased cardiovascular and overall mortality compared with those on placebo (*p* for interaction 0.019) (155). Ticagrelor or prasugrel may lower platelet reactivity more efficiently in comparison to clopidogrel, and this effect seems to be independent from renal function (156, 157).

In summary, the use of anticoagulants and antiplatelet medications has to be rationalized and balanced due to the increased risk of bleeding in CKD patients.

## Conclusion

As kidney function declines cardiovascular system develops various pathologies. Indeed cardiovascular morbidity and mortality has been a focus for nephrologists for decades. However, chronic kidney disease (CKD) affects brain function and structure as well. Kidney and brain disorders share common pathophysiologic mechanisms. Common risk factor modifications include treatment of high blood pressure in order to prevent hypertension induced chronic kidney disease, stroke and silent brain infarction. Brain imaging tests could be routinely used in patients with CKD to detect silent brain infarctions, white matter lesions and cerebral microbleeds as these asymptomatic conditions may portend fatal consequences.

In summary, CKD has a direct damaging effect on the brain, and the mechanisms are complex and further investigations are needed. As the renal and cerebral disorders are inextricably linked, their management should include a close collaboration between nephrologists and neurologists.

## Author Contributions

All authors contributed significantly to this work and supported the publication of the manuscript. MM, UC, MJ, and AV devised the research plan. MM, UC, MJ, and AV wrote the manuscript. MM and MJ modified and polished the manuscript.

## Conflict of Interest

The authors declare that the research was conducted in the absence of any commercial or financial relationships that could be construed as a potential conflict of interest.
